# rs12512631 on the Group Specific Complement (Vitamin D-Binding Protein *GC*) Implicated in Melanoma Susceptibility

**DOI:** 10.1371/journal.pone.0059607

**Published:** 2013-03-27

**Authors:** Maria Peña-Chilet, Maider Ibarrola-Villava, Manuel Martin-González, Marta Feito, Cristina Gomez-Fernandez, Dolores Planelles, Gregorio Carretero, Ana Lluch, Eduardo Nagore, Gloria Ribas

**Affiliations:** 1 Medical Oncology and Haematology Unit, Health Research Institute INCLIVA, Valencia, Spain; 2 Dermatology Department, Ramon y Cajal Hospital, Madrid, Spain; 3 Dermatology Department, La Paz University Hospital, Madrid, Spain; 4 Laboratory of Histocompatibility-Molecular Biology, Centre for Blood Transfusion, Valencia, Spain; 5 Dermatology Department, Doctor Negrín Hospital, Las Palmas de Gran Canaria, Spain; 6 Dermatology Department, Instituto Valenciano de Oncología, Valencia, Spain; University of Illinois at Chicago, United States of America

## Abstract

**Background:**

Solar radiation should be avoided in melanoma patients. Nevertheless, this is the main means by which the body produces vitamin D. Evidence suggests a protective role against cancer for vitamin D. Since vitamin D performs its function by binding the receptor encoded by the vitamin D-receptor gene (*VDR)*, most studies have focused on polymorphisms (SNPs) within this gene. However, the gene encoding the vitamin D-binding protein (*GC)* appears in recent studies as a major player in the role of a serum vitamin D level regulator and in Cutaneous Melanoma (CM) predisposition.

**Methods:**

We performed a case-control study of 12 polymorphisms on *GC* and 9 on *VDR* among 530 cases and 314 controls from Spanish population.

**Results:**

We found association between SNP rs12512631, located 3′downstream of *GC*, and risk of CM that seems to fit a dominant model (OR 1.63 95%CI 1.23–2.17 p-value 7×10^−4^). This association remained Bonferroni’s correction and after adjustment for potential confounders (p-value 3×10^−3^) and even after increasing the sample size to 1729 individuals (p-value 0.0129). Moreover, we confirmed evidence of an association between CM susceptibility and the linkage disequilibrium block marked by tag-SNP rs222016 (p-value 0.032). This block covers the *GC* intron 1 region, with probable regulatory functions.

**Conclusion:**

To our knowledge, this is the first vitamin D pathway-related polymorphism study in melanoma risk conducted in the Spanish population. Furthermore, we show an association between polymorphisms in *GC* and melanoma risk, confirming recent studies in different populations.

## Introduction

Cutaneous Melanoma (CM) is caused by the malignant transformation of melanocytes, pigment producer cells, located within epidermal basal cells. These cells produce melanin as a response to UV radiation. Melanoma incidence has been increasing at higher rates than any other malignant tumour in recent decades, and it causes the greatest number of skin-cancer-related deaths worldwide [1]. The main reason for the recent rise in CM incidence is attributed mainly to increased intermittent exposure to UV radiation.

The data about whether sun exposure in Caucasian populations results in health risks or benefits is controversial. Solar radiation is the main source of vitamin D synthesis in humans; however, uncontrolled and intensive sun exposure is dangerous to health and contributes to the development of melanoma [2], [3]. Given that occupational sun exposure has been reported to protect against melanoma, that solar radiation is the main means by which the body produces vitamin D, and that vitamin D is reported to have anti-proliferative effects in melanoma cells, interest has arisen over a possible role for vitamin D in melanoma prevention [4].

The most studied gene in the vitamin D pathway is the vitamin D receptor gene (*VDR)* located on chromosome 12. The gene has 11 exons and encodes the receptor of the calcitriol form of vitamin D. This receptor is a nuclear transcription and regulating factor that belongs to the steroid hormone superfamily of receptors. Nuclear receptors integrate hormonal, dietary, and other extracellular signals into cell fate decisions via regulation of gene expression and repression of a host of common gene targets. Moreover, many studies have addressed the polymorphisms of the vitamin D receptor in several cancers including ovarian carcinoma [5], breast cancer [6], colorectal cancer [7], non-Hodgkin lymphoma [8], renal cancer [9], oral squamous cell carcinoma [10], esophageal adenocarcinoma [11], non-small cell lung cancer [12], prostate cancer [13], and melanoma [14], [15].

Most recently, genome-wide association studies (GWAS) reported that a SNP in the *GC* gene is associated with serum levels. This gene encodes for the group-specific component or VDBP (vitamin D-binding protein), with the additional probable involvement of genes related to the production of the active form of vitamin D [16]. VDBP belongs to the albumin family, together with human serum albumin and alpha-fetoprotein. Located on chromosome 4q11–q13, the *GC* gene is 42.5 kb long and contains 13 exons. At least six non-synonymous SNPs are described, two of them with common frequency (rs7041 and rs4588). The VDBP greatly facilitates vitamin D actions by carrying vitamin D metabolites to various sites of action, while polymorphic VDBP proteins differ in their affinity for 1,25(OH)2 D metabolite [17]. VDBP has been related to multiple sclerosis [18], its association with various lung diseases including asthma, chronic obstructive pulmonary disease and tuberculosis has also been studied [19]. Recent studies, including GWAS, have shown that allelic variation in the *GC* gene is associated with both VDBP and serum 25(OH)D concentrations, as well as a higher affinity of the VDBP to vitamin D metabolites [16], [20].

Despite the large number of studies evaluating the association between *VDR* variants and CM, the conclusions on its role in the aetiology are still indecisive. The association between *GC* variants and vitamin D levels in plasma has already been tested and proved [16], [20], but only one study supports the association between polymorphisms on *GC* and CM [21]. In the present study, we show a comprehensive analysis of *VDR* and *GC* association with CM using data from a wide case-control study (530 melanoma cases and 314 controls) in a Spanish population. Data from *VDR* have been enlarged in sample size and density of SNPs from previous results [22]. We consider that the present study increases our knowledge of the relationship between vitamin D levels and CM predisposition in a Southern European country such as Spain, where sun incidence is higher than in northern European countries.

## Materials and Methods

### Ethics Statement

All participants gave written informed consent for this study. The study was approved by the Ethics Committee of Gregorio Marañón University General Hospital.

### Study Subjects and Data Collection

A total of 530 sporadic CM patients were recruited from 1^st^ September 2004 to the present, at the departments of Dermatology of three different hospitals in Madrid: Gregorio Marañón University General Hospital, La Paz University Hospital and Ramón y Cajal University Hospital. A total of 314 volunteer cancer-free control samples, frequency matched to cases by sex and age in ten-year categories, were recruited from the Madrid College of Lawyers, the National Cancer Research Centre (CNIO) and from the Gregorio Marañón University General Hospital (Table 1). All participants were non-related Caucasians of Spanish origin, with the same ethnic background [23].

**Table 1:** Sample distribution.

Phase	Institution of origin for samples	Cases N (%)	Controls N (%)
Set I	Gregorio Marañon Hospital	194 (36.61)	94 (29.94)
	Ramón y Cajal Hospital	172 (32.45)	0 (0.00)
	La Paz Hospital	120 (22.64)	10 (3.18)
	Madrid College of Lawyers/CNIO	0 (0.00)	237 (75.48)
	**Total**	**530**	**314**
Set II	Valencian Institute of Oncology (IVO)	344 (66.80)	158 (42.70)
	Dr. Negrín Hospital	211 (40.97)	172 (46.49)
	**Total**	**515**	**370**
Total		1045	684

CNIO, National Cancer Research Centre, Madrid.

All hospital participants in Set I are from Madrid. The IVO is in Valencia and Dr Negrín Hospital is located in Las Palmas de Gran Canaria, on Canary Islands, as are the populations sampled for each hospital.

The percentage is calculated from the total of cases or controls, respectively, for each phase.

A standardized questionnaire was used to collect information on pigmentation characteristics such as eye, hair and skin colour, number of naevi, presence of lentigines, sun exposure habits, and personal and family history of melanoma, cancer or any other skin disease. Each individual questionnaire has been guided by an expert clinician or a trained nurse. For cases only, tumour characteristics were added and medical data were obtained via medical exploration, those patients with acral or multiple melanoma were excluded from the study, as well as control individuals with suspected personal or family history of melanoma. We selected the following variables: age at diagnosis, sex, eye colour categorized as blue, grey and light green (light eye colour), hazel, light brown, brown and black (dark eye colour), hair was grouped as light colour (very light blonde and red-haired) and dark colour (light brown, medium brown and black) the skin colour stratification was made by the clinicians implicated in the project as very light and never tans, light, medium and dark, however to compare between cases and controls we only show two main categories, fair skin color (very light and never tans and light) and dark skin color (medium and dark), number of naevi (less than 25, between 25–50, between 50 and 100 and more than 100), presence of solar lentigines (yes, no and only on shoulders) and childhood sunburn events (categorization of these variables as well as the distribution of the Spanish population sampled are shown in Table 2).

**Table 2:** Classification of the Spanish samples studied by age, sex and phenotype.

Characteristic	Controls (N = 314) n (%)	Cases (N = 530) n (%)	P-Fisher	OR (95% CI)	P-value*
Age at diagnosis (years)			0.223	1.23 (0.90–1.69)	0.201
<Mean	126 (40.13)	233 (43.96)			
≥Mean	97 (30.89)	226 (42.64)			
Unknown	91 (28.98)	71 (13.40)			
Mean (SD)	52.94 (16.02)	52.32 (15.96)			
Sex			0.667	0.96 (0.72–1.27)	0.751
Men	143 (45.54)	237 (44.72)			
Women	166 (52.87)	288 (54.34)			
Unknown	5 (1.59)	5 (0.94)			
Eye colour			**2.67**×**10** ^−**07**^	**2.44 (1.77–3.36)**	**4.96**×**10** ^−**08**^
Dark eye colour	235 (74.84)	307 (57.92)			
Light eye colour	68 (21.66)	217 (40.94)			
Unknown	11 (3.50)	6 (1.13)			
Hair colour			**1.13**×**10** ^−**08**^	**3.37 (2.15–5.28)**	**8.43**×**10** ^−**08**^
Brown/Black	284 (90.45)	398 (75.09)			
Blond/Red	25 (7.96)	122 (23.02)			
Unknown	5 (1.59)	10 (1.89)			
Skin colour			0.076	1.16 (0.83–1.49)	0.452
Dark skin colour	132 (42.04)	213 (40.19)			
Fair skin colour	171 (54.46)	308 (58.11)			
Unknown	11 (3.50)	9 (1.70)			
Lentigines			**1.54**×**10** ^−**14**^	**3.39 (2.50–4.59)**	**3.86**×**10** ^−**15**^
No	157 (50.00)	142(26.79)			
Yes	124 (39.49)	380(71.70)			
Unknown	33 (10.51)	8(1.51)			
Number of Naevi			0.100	1.53 (0.98–2.40)	0.059
<50	245 (78.03)	441(83.21)			
≥50	30 (9.55)	83(15.66)			
Unknown	39 (12.42)	6(1.13)			
Childhood sunburn			**1.21**×**10** ^−**14**^	**6.30 (4.52–8.77)**	**1.11**×**10** ^−**27**^
No	206 (65.61)	157 (29.62)			
Yes	70 (22.29)	336 (63.40)			
Unknown	38 (12.10)	37 (6.98)			

* Fisher's exact test. P-value, excluding unknown values.

Bold denotes statistically significant results.

SD, standard deviation.

We used an additional pool of samples to enlarge the sample size. Samples were obtained from 334 CM patients and 158 control individuals from the Valencian Institute of Oncology (IVO) and 171 cases and 212 controls from Dr Negrín Hospital, Las Palmas de Gran Canaria. A total set of 515 cases and 370 control individuals (Table 1).

Genomic DNA from cases and controls was isolated from peripheral blood lymphocytes and diluted to a final solution of 50 ng/µl using the traditional saline method or the DNAzol procedure (Invitrogen, Eugene, OR, USA). DNA concentration was quantified in samples using Quant-iT PicoGreen dsDNA Reagent (Invitrogen, Eugene, OR, USA). Further concentration measures were obtained using a Nanodrop 2000 spectrophotometer. Genomic DNA was amplified using the GenomiPhi DNA Amplification Kit (GE Healthcare Bio-Sciences AB, Uppsala, Sweden).

### SNP Selection

We selected tag-SNPs from vitamin D metabolism-related genes, *GC* and *VDR*. These SNPs were chosen using either previous literature information or the HapMap International Project [24] by means of the HaploView v4.2 software forcing Tag-SNPs from the European_CEU subset of data. We selected marker SNPs with a minor allele frequency (MAF) higher than 0.05, with a Hardy-Weinberg equilibrium p-value cutoff of 0.001, we have set an r^2^ threshold of 0.8. Four linkage disequilibrium (LD) blocks were obtained using HapMap_CEU data for *GC* and seven blocks for *VDR*. A total of 21 SNPs were included (12 SNPs belonging to *GC* and 9 to *VDR*). Public databases were used to collect additional information about SNPs and genes: NCBI http://www.ncbi.nlm.nih.gov and Ensembl http://www.ensembl.org. Details such as MIM code, location, encoded protein, context sequence, nucleotide changes, MAF for HapMap_CEU and HapMap_TSI (based on a sample population from Northern Europe and Tuscany Italy respectively) and calculated MAF for both cases and controls of all the SNPs studied are provided in Table S1 and in Table 3.

**Table 3:** Allelic frequencies in cases and controls in spanish population for the GC gene SNPs studied.

Gene	SNP	MAF HapMap_CEU	MAF HapMap_TSI	MAF Controls	MAF Cases	p HWE	Allelic p-value
*GC*	rs12512631	0.35	0.32	0.31	0.37	0.027	**0.0196**
	rs222049	0.08	0.10	0.09	0.08	0.341	0.324
	rs2282679	0.26	0.26	0.30	0.31	0.220	0.734
	rs705119	0.43	0.46	0.43	0.44	0.299	0.571
	rs4588	0.27	0.26	0.30	0.30	0.652	0.991
	rs7041	0.43	0.47	0.43	0.46	0.365	0.337
	rs188812	0.10	0.10	0.08	0.10	0.922	0.254
	rs222016	0.16	0.14	0.11	0.14	0.515	0.103
	rs1155563	0.33	0.23	0.30	0.30	0.444	0.963
	rs1352844	0.14	0.13	0.10	0.11	0.976	0.912
	rs1352845	0.21	0.17	0.14	0.15	0.464	0.577
	rs3733359	0.06	0.04	0.03	0.04	0.573	0.729
*VDR*	rs11574143	0.10	0.15	0.10	0.10	0.240	0.918
	rs739837	0.43	0.40	0.49	0.49	0.243	0.779
	rs731236	0.43	0.41	0.39	0.38	0.884	0.639
	rs2228570	0.41	0.38	0.34	0.34	0.309	0.848
	rs4334089	0.27	0.27	0.30	0.27	0.835	0.190
	rs4237855	0.42	0.35	0.48	0.47	**1.09**×**10** ^−**12**^	0.760
	rs7299460	0.31	0.34	0.34	0.31	0.666	0.374
	rs4760658	0.30	0.36	0.32	0.34	0.461	0.273
	rs4516035	0.38	0.44	0.40	0.41	0.531	0.593

Bold marks statistically significant results.

p HWE refers to the p value for Pearson's goodness-of-fit test for deviation from Hardy Weinberg's equilibrium among controls.

MAF, minor allele frequency.

p-value for Pearson's goodness-of-fit chi-square between cases and controls MAF.

HapMap_CEU and HapMap_TSI refers to Caucassian European and Tuscany Italian populations respectively, obtained from HapMap database.

### Genotyping Assays

Genotyping was carried out using Kaspar technology (KBiosciences, Hoddesdon, UK). The PCR was performed in a reaction volume of 4 µl containing about 10 ng of genomic DNA, a final concentration of 4× New Kaspar Reaction Mix, and 12 µM of each Kaspar primer.

The PCR assays were performed according to the manufacturer’s instructions. The genotype of each sample was determined by measuring final allele-specific fluorescence in the ABI Prism 7900 HT Detection System, using the SDS 2.3 software for allelic discrimination (Applied Biosystems, Foster City, USA).

As a quality control measure, we included one non-template sample and one sample duplicate per 96-well plate (a total of four per 384-well plate used). Genotypes were provided automatically by the software and were confirmed manually by two different laboratory personnel.

### Statistical Analyses

Analyses were performed using SPSS v17 (SPSS, Chicago, IL, USA). All p-values were two-sided, and those less than 0.05 were considered statistically significant. We analysed the haplotypes using Haploview v4.2 software, we obtained χ^2^ values by performing a linkage case-control test as described previously [25]. Bonferroni’s correction was used as the method of adjustment for multiple comparisons. For all polymorphisms studied, Fisher's exact test was used both to test for deviations from HWE among controls, and to compare differences in the MAF distributions between cases and controls. We rejected HWE when p-values were lower than 0.0024 according to Bonferroni’s correction for 21 comparisons. In order to assess associations between genotypes, haplotypes and CM risk and between SNPs and each phenotypic characteristic, several analyses were performed. Genotype-related odds ratios (ORs), their corresponding 95% confidence intervals (CIs) and associated p-values were estimated via unconditional logistic regression. This was done for each model: genotypic, dominant and recessive. Same analyses were conducted between SNPs and each phenotypic characteristic. The power of the significant results was obtained using POWER v3.0 software (available at http://dceg.cancer.gov/tools/design/power), with the sample size of this study we are able to obtain a power of 80% as from OR value of 1.49. Odds ratios were then adjusted for known and suspected melanoma risk factors (eye, hair and skin colour, number of naevi, lentigines, and childhood sunburns) in order to assess the potential confounding effects by multivariate logistic regression We use as potential confounders all phenotypic traits that show differences between cases and controls (Table 2), however we added number of naevi and skin color in this study because both are well recognized risk factor for melanoma predisposition [26], [27].

For the second set of samples we obtained genotype-related ORs for genotypic, dominant and recessive models, their corresponding 95% CIs and associated p-values taking into account only SNPs that gave interesting results. These statistics were estimated using unconditional logistic regression.

### Functional Analyses

To study the functional implications of SNPs, we used the Pupasuite3.1 software available online at http://pupasuite.bioinfo.cipf.es. We analysed all SNPs on the *GC* gene with a minor allele frequency higher than 0.05 provided by HapMap (a total of 40 SNPs, provided upon request). We searched for all non-synonymous SNPs, candidate transcription factor (TF) binding sites (through TRANSFAC, JASPAR and ORegAnno), low-flexibility promoter regions with a minimum length of 10 bp, highly conserved regions, splice sites created or disrupted by SNPs, and the presence of miRNA targets. Search criteria were provided at the aforementioned website.

## Results

### Allelic Distribution of Polymorphisms and Association with CM Risk

After applying Bonferroni’s correction, one SNP on *VDR* was out of HWE, rs4237855 (p-value 1.09×10^−12^), and was removed from further analyses. The remaining SNPs complied with HWE. We confirmed that our control population has allele frequencies similar to the HapMap_CEU or HapMap_TSI ones.

Based on unadjusted p-values, we observed evidence of differences between cases and controls in MAF for SNP rs12512631 on the *GC* gene (p-value 0.0196) which codes for the vitamin D transporter protein. We did not observe differences in the MAF for any other SNP. (Table 3).

Allele frequencies for SNPs (on HapMap_CEU, HapMap_TSI and genotyped cases and controls), p-values for their comparison between 530 CM cases and 314 control individuals, along with p-values for the test of departure from HWE among controls are detailed in Table 3.

### Association between Genotypes and Melanoma Risk

The implication of these vitamin D-related genes in melanoma was investigated further by comparing the genotypic distributions of all the SNPs studied. The estimated ORs and associated p-value*s* are shown in Table 4. This was also done for dominant and for recessive models. Relevant results as well as the values adjusted by phenotypic characteristics are shown in Table 5.

**Table 4:** Genotypic distribution among cases and controls in a Spanish population.

		Controls (N = 314) n (%)			Cases (N = 530) n (%)				
Gene	SNP	Major homozygotes	Heterozygotes	Minor homozygotes	Major homozygotes	Heterozygotes	Minor homozygotes	OR (95% CI)	p-value
*GC*	rs12512631	154 (49.04)	115 (36.62)	38 (12.10)	199 (37.55)	261 (49.25)	61 (11.51)	1.29 (1.04–1.60)	**0.0190**
	rs222049	240 (76.43)	50 (15.92)	1 (0.32)	441 (83.21)	74 13.96)	2 (0.38)	0.83 (0.57–1.20)	0.3150
	rs705119	102 (32.48)	136 (43.31)	58 (18.47)	152 (28.68)	276 (52.08)	90 (16.98)	1.06 (0.86–1.31)	0.5649
	rs4588	154 (49.04)	128 (40.76)	30 (9.55)	248 (46.79)	229 (43.21)	42 (7.92)	1.00 (0.81–1.25)	0.9910
	rs7041	101 (32.17)	140 (44.59)	60 (19.11)	145 (27.36)	271 (51.13)	100 (18.87)	1.11 (0.90–1.36)	0.3333
	rs188812	261 (83.12)	44 (14.01)	2 (0.64)	425 (80.19)	97 (18.30)	1 (0.19)	1.25 (0.86–1.80)	0.2422
	rs222016	236 (75.16)	58 (18.47)	5 (1.59)	368 (69.43)	127 (23.96)	8 (1.51)	1.30 (0.95–1.77)	0.1020
	rs1155563	150 (47.77)	121 (38.54)	30 (9.55)	252 (47.55)	217 (40.99)	45 (8.61)	0.99 (0.79–1.25)	0.9633
	rs1352844	221 (70.38)	51 (16.24)	3 (0.96)	416 (78.49)	93 (17.55)	8 (1.51)	1.02 (0.73–1.42)	0.9137
	rs1352845	204 (64.97)	70 (22.29)	4 (1.27)	365 (68.87)	138 (26.04)	8 (1.51)	1.09 (0.81–1.48)	0.5675
	rs3733359	254 (80.89)	18 (5.73)	0 (0)	483 (91.13)	38 (7.17)	0 (0)	1.10 (0.62–1.97)	0.7400
*VDR*	rs11574143	238 (75.80)	55 (17.52)	1 (0.32)	383 (72.26)	88 (16.60)	1 (0.19)	0.98 (0.68–1.41)	0.9143
	rs739837	74 (23.57)	160 (50.96)	66 (21.02)	121 (22.83)	258 (48.68)	115 (21.70)	0.97 (0.79–1.19)	0.7731
	rs731236	109 (34.71)	141 (44.90)	44 (14.01)	186 (35.09)	248 (46.86)	64 (12.09)	0.95 (0.76–1.18)	0.6325
	rs2228570	140 (44.59)	130 (41.40)	39 (12.42)	217 (40.94)	225 (42.52)	58 (11.01)	1.02 (0.82–1.27)	0.8502
	rs4334089	138 (43.95)	121 (38.54)	25 (7.96)	261 (49.25)	192 (36.23)	36 (6.79)	0.86 (0.68–1.08)	0.1898
	rs4237855	102 (32.48)	76 (24.20)	91 (28.98)	165 (31.13)	129 (24.34)	140 (26.42)	0.98 (0.81–1.17)	0.7977
	rs7299460	131 (41.72)	137 (43.63)	32 (10.20)	236 (44.53)	224 (42.26)	47 (8.87)	0.90 (0.73–1.13)	0.3680
	rs4760658	136 (43.31)	134 (42.68)	27 (8.60)	225 (42.45)	212 (40)	67 (12.64)	1.13 (0.91–1.39)	0.2795
	rs4516035	106 (33.76)	149 (47.45)	45 (14.33)	183 (34.53)	228 (43.02)	94 (17.74)	1.06 (0.86–1.29)	0.5980

Heterozygotes and minor homozygotes individuals count for cases and controls and their percentages are calculated among the total of samples including fails.

OR (CI 95%) means Odds Ratio and its 95% confidence interval in parentheses. OR and p values are calculated via unconditional logistic regression considering differences between cases and controls genotypes.

Bold denotes statistically significant results considering as significant p values lower than 0.05.

**Table 5:** Genotypic analyses of SNPs with Cutaneous Melanoma risk association.

		Non-adjusted		Adjusted*		Enlarged sample	
SNP	Statistical model	OR (CI 95%)	p-value	OR (CI 95%)	p-value	OR (CI 95%)	p-value
rs12512631	Genotypic	1.29 (1.04–1.60)	**0.019**	1.32 (1.01–1.72)	**0.041**	1.11 (0.96–1.28)	0.153
	Dominant	1.63 (1.23–2.17)	**7**×**10** ^−**4**^	1.71 (1.20–2.45)	**3**×**10** ^−**3**^	1.28 (1.05–1.56)	**0.012**
	Recessive	0.94 (0.61–1.45)	0.774	0.94 (0.55–1.60)	0.809	0.90 (0.68–1.19)	0.458
rs222016	Genotypic	1.30 (0.95–1.77)	0.102	1.23 (0.83–1.82)	0.312	1.11 (0.92–1.35)	0.277
	Dominant	1.37 (0.98–1.93)	0.068	1.30 (0.85–1.98)	0.222	1.22 (0.97–1.52)	0.083
	Recessive	0.95 (0.31–2.93)	0.929	0.65 (0.14–3.09)	0.586	0.65 (0.35–1.20)	0.168

Bold denotes statistically significant results.

OR (CI 95%) means Odds Ratio and its 95% confidence interval.

* Adjusted for eye colour, hair colour, skin colour, lentigines, number of naevi and childhood sunburn.

The enlarged sample makes a total of 684 controls and 1045 cases.

One SNP was found to be associated with CM susceptibility risk, rs12512631 on the *GC* gene (OR 1.29 95%CI: 1.04–1.60; p*-*value 0.0190) (Table 4). Moreover, the association between rs12512631 and CM risk was highly significant in the dominant model (OR 1.63 95% CI: 1.23–2.17; p-value 7×10^−4^) (Table 5). This significance remained after Bonferroni’s correction for multiple testing.

A trend to significance was found for rs222016 (OR 1.23 95% CI: 0.83–1.82; p-value 0.102). Furthermore, when performing the statistical analyses considering dominant model, we obtained borderline significance (OR 1.37, 95% CI: 0.98–1.93; p*-*value 0.068). No association remained statistically significant for any other SNP.

To assess the independence of risk factors associated with CM, we performed a multivariate analysis that took into account phenotypic risk factors such as eye, skin and hair colour, number of naevi, lentigines and childhood sunburn events, along with candidate SNPs. The associated SNP, rs12512631, maintained its significance for both genotypic (p-value 0.041) and dominant models (p-value 0.003), whereas there was no further trend to significance for rs222016 (see adjusted values in Table 5).

We increased the population sample and performed the association analyses for the two significant CM-associated SNPs (rs12512631 and rs222016). We studied a total of 1045 melanoma patients and 684 control individuals, and the statistical significance of rs12512631 remained (OR 1.28 95% CI: 1.05–1.56, p-value 0.013). This association continued to be significant after Bonferroni’s correction for two comparisons (p-value threshold of 0.025) (Table 5).

### Associations between Genotypes and Phenotypic Characteristics

We assessed whether SNPs from the *GC* and *VDR* genes were associated with various phenotypic characteristics. The SNP rs3733359, located on the 5′UTR of the *GC* gene, showed a significant association with dark skin colour (OR 0.53, 95% CI: 0.30–0.94, p-value 0.023). We also observed a weak significant association for the 3′UTR SNP rs739837 of the *VDR* gene with fair skin colour (p-value 0.048). We found strong association between non-synonymous SNP rs2228570, located within exon 4 of the *VDR* gene, and absence of childhood sunburn (OR 0.065 95% CI 0.49–0.86, p-value 0.003). No more evidence of associations was found.

### Haplotype Analysis and Association with Melanoma Risk

**Figure 1:**
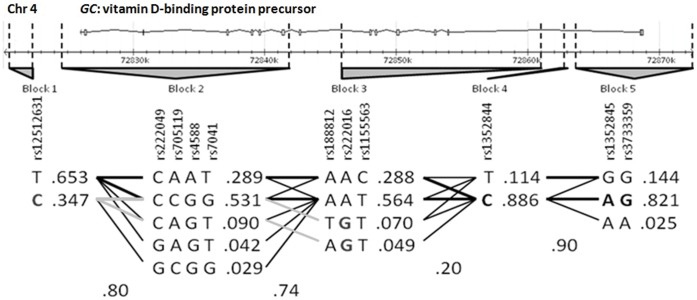
Haplotype distribution within *GC* gene according to Tag-SNPs selected by Haploview v4.2. Genotyped SNPs are indicated by their rs#. LD Blocks are shown in gray. *GC* gene chromosomic location and ideogram in reverse orientation appears on the top of the figure, where boxes represent exons and horizontal black lines introns. Bold denotes associated risk alleles, and light grey lines indicate connected haplotypes.

We performed haplotype analyses using the 11 tag-SNPs in *GC* (the gene on which we found an associated SNP) selected previously from HapMap data. These SNPs were organized into five independent blocks according to the Haploview program for the use of data from a European subset of samples (HapMap_CEU). Block 1 included rs12512631 in a 3′downstream region of *GC*. Block 2 is represented by one SNP located 3′downstream and three SNPs within the 3′end of the gene, two of them corresponding to exon 9. Blocks 3, 4 and 5 contained six SNPs from various intron regions, including intron 1, and SNP with rs3733359, located on 5′UTR. Block 5 represented the most likely promoter region with consensus sequences for TF binding sites. Data analysis was done according to the block structures detailed in Figure 1. The case-control analysis of the haplotype distribution revealed a statistical association with melanoma susceptibility on two SNPs, rs12512631 (p-value 9×10^−3^), and rs222016 (p-value 0.031), representing Blocks 1 and 3, respectively (Table S2). These data were coherent with the associations described above.

### Functional Implication

To assess for possible functional implications of polymorphisms, we carried out a prioritization of SNPs based on functional properties using the Pupasuite 3.1 software. Results showed non-synonymous changes of aminoacid on rs4588 (K436T) and rs7041 (E416D). TRANSFAC revealed three SNPs located in consensus sequence affecting the TF binding sites; rs4588 FOXJ2, rs7041 HNF-1 and rs222014 E2F-1 (located within intron 3 and grouped on block 3 with tag-SNP rs222016). None of the other SNPs seemed to have any additional functionality.

## Discussion

In this case-control study, we have detected for the first time statistical evidence suggesting that rs12512631 on *GC* is associated with risk for CM. *GC* is an essential gene in the vitamin D pathway since it codifies for VDBP, the transporter of all the intermediate and final forms of vitamin D. Variants in this gene may modulate protein expression or activity of this protein and, therefore may affect vitamin D synthesis and distribution. There are few studies of *GC* polymorphisms, however, to our knowledge all of them are related to the serum vitamin D levels in diseases such as CM, prostate and colorectal cancer. In these, a clear association was found between *GC* polymorphisms and circulating vitamin D levels, but the association between polymorphisms and the disease itself was not at all clear [21], [28], [29], [30]. Shen et al. reported that one allele of the *GC* gene may be a risk factor for chronic obstructive pulmonary disease [31], and Abbas et al. observed a lower breast cancer risk associated with the *Gc2–2* allele, independent of vitamin D levels [32]. Recently an independent GWAS on vitamin D levels, with further validation on CM patients, has pointed again to the *GC* gene as a candidate for melanoma susceptibility when vitamin D levels are taken into consideration [16], [21].

Our study suggests that the 3′downstream region of the *GC* gene, marked through the SNP rs12512631, is associated with CM risk. Furthermore, evidence of association was suggested by the tag-SNP rs222016, which marks a large disequilibrium block within the gene, including intron 1. SNP rs222016 is in LD with SNP rs222014, which could have functional implications through the disruption of a transcription factor element (E2F-1), but we cannot discard other mechanisms such as alternative splicing signalling. Previous studies have shown an association between tag-SNP rs12512631 and susceptibility to prostate and colorectal cancer, which supports our results [28], [29].

We did not observe any evidence of association between CM and *VDR* variants overall, in accordance with our previous results [22]. Some other controversial results are reported in the literature. A review by Köstner indicates only one variant in *VDR* associated with CM risk, Fok1 (rs2228570) [33], but a meta-analysis of various studies revealed that the only variant that presents solid evidence is Bsm1 (not considered in our study) and remarked on the need to adjust by phenotype or environmental characteristics [34]. Gapska et al. found an association only when analyzing the haplotypes, but not the variants themselves [14]. More recent studies have not detected an association with CM risk on the *VDR* variants unless adjusting by vitamin D levels [15], [35], [36]. This last situation might explain why we have not found a significant association; vitamin D levels were not taken into account.

The strength of this study is the homogeneity of the Spanish population sample and the ability to control for established risk factors for CM through a structured questionnaire. We recognize, however, some potential for misclassification of phenotypic characteristics due to the subjective nature of the phenotypic attributes considered. Controls participated on a volunteer basis, which may have introduced some selection bias.

We show a comprehensive study of genetic variation on the vitamin D pathway genes *VDR* (vitamin D receptor) and *GC* (vitamin D transporter), and examine their putative role in CM susceptibility. We observed statistically significant results for CM susceptibility with two variants in the *GC* gene, but none in the *VDR* gene. One of these associations remained significant after correction for multiple testing, SNP rs12512631. This association suggests that *GC* may also play a role in modulating the susceptibility to CM.

We encourage replication of these findings in independent studies since the *GC* gene may well be a new marker for CM predisposition.

## Supporting Information

Table S1 **SNPs on** ***GC and VDR*** **genes considered in this study.** *GC* refers to Vitamin D binding protein gene; *VDR* refers to Vitamin D receptor gene. Bold in sequence context denotes nucleotide change. Location is described considering as the first Exon 1 of consensus sequence. DWST means downstream, UTR means untranscribed region and UPST means upstream.
(DOCX)

Table S2 **Case-control study based on haplotypes in the** ***GC*** **gene conducted in Spanish population.** Bold denotes statistically significant results. Italic Haplotypes on each block mark the risk haplotype. The Marker number indicates the order of the tag-SNP on the gene. LD Block means linkage disequilibrium block. Frequencies are calculated for the association alleles of each haplotype among 530 cases and 314 controls.
(DOCX)
