# Supplementary material for: rs12512631 on the Group Specific Complement (Vitamin D-Binding Protein GC) Implicated in Melanoma Susceptibility
Source: PLoS One. 2013 Mar 27;8(3):e59607. doi: 10.1371/journal.pone.0059607 (PMC3609832; doi:10.1371/journal.pone.0059607)
Supplement: Table S1 — SNPs on GC and VDR genes considered in this study. GC refers to Vitamin D binding protein gene; VDR refers to Vitamin D receptor gene. Bold in sequence context denotes nucleotide change. Location is described considering as the first Exon 1 of consensus sequence. DWST means downstream, UTR means untranscribed region and UPST means upstream. (DOCX) [file pone.0059607.s001.docx]

| **Table S1.** SNPs on *GC and VDR* genes considered in this study. | | | | | | | | |
| --- | --- | --- | --- | --- | --- | --- | --- | --- |
| **Gene symbol (MIM ID)** | **Also known as** | **Gene location** | **SNP** | **Location** | **Aminoacid change** | **Major allele** | **Minor allele** | **Sequence context** |
|  |  |  |  |  |  |  |  |  |
| *GC* (139200) | NM_000583.2 | Chr4q11-q13 | rs12512631 | 3' DWST |  | T | C | GTTGTCATGGAAGAAACAATTAGATG**[C/T]**TTTCTTGGCCTAATAGAGAGAAAAG |
|  | *VDBP* |  | rs222049 | 3' DWST |  | C | G | CTCTAAGTCACAGAGTTAGCAAATAA**[C/G]**AAAACAAGGCCTTAGTTTTCAATTG |
|  | *VDBG* |  | rs2282679 | intron11 |  | A | C | AATCTCTGTCTCTTAATTATCTCACA**[A/C]**AGCCAGGTATTTTTTATTGTTAGCTT |
|  | *GRD3* |  | rs705119 | intron 11 |  | C | A | GAGGTAGAGATTTGGAGATTAAAAGC**[A/C]**TAAGCATTGATGAAAACAGTTGAGA |
|  | *DBP* |  | rs4588 | exon 11 | K436T | G | A | AGCAAAATTGCCTGATGCCACACCCA**[A/C]**GGAACTGGCAAAGCTGGTTAACAAG |
|  |  |  | rs7041 | exon 11 | E416D | G | T | GAGCGACTAAAAGCAAAATTGCCTGA**[G/T]**GCCACACCCACGGAACTGGCAAAGC |
|  |  |  | rs188812 | intron 6 |  | A | T | GTGCAGAAGGGAAATGTGGGTGTGGA**[A/T]**CCTCCAAATAGAGTCCCTACTGGGG |
|  |  |  | rs222016 | intron 2 |  | A | G | GGATTAAGGATTAACCTCCATGCTGA**[A/G]**TCAAAGTTACCTCACACTCAGTTTT |
|  |  |  | rs1155563 | intron 1 |  | T | C | TGTGCTCTTGCTATTGTATTTTTTAA**[C/T]**AGATAAAAAAATCACAGTTATTTCC |
|  |  |  | rs1352844 | intron 1 |  | C | T | TGTAGTGTTTCTCAGAACCTTTAGGA**[C/T]**GACAAAGGCATGAATTGTGAAACCA |
|  |  |  | rs1352845 | intron 1 |  | A | G | AGAACCTTTAGGACGACAAAGGCATG**[A/G]**ATTGTGAAACCACCAAGAAGGAATA |
|  |  |  | rs3733359 | 5' UTR |  | G | A | GGCTACCACTTTTACATGGTCACCTA**[C/T]**AGGAGAGAGGAGGTGCTGCAAGACT |
| *VDR* (601769) | NM_000376.2 | Chr12q13 | rs11574143 | 3' DWST |  | G | A | TGTGTCTGCCATTAGAGATGGCGGCT**[A/G]**TGCCAGTGACCTGGAGGATTACAAA |
|  | *NR1\|1* |  | rs739837 | 3' UTR |  | G | T | CCTCAACATCAGTCAGCAGCCACTTA**[G/T]**GCAGCGGTGGAGGCATCTCTGGGCA |
|  |  |  | rs731236 | exon11 | I352 | T | C | CCTGGGGTGCAGGACGCCGCGCTGAT**[C/T]**GAGGCCATCCAGGACCGCCTGTCCA |
|  |  |  | rs2228570 | exon4 | M162T | A | G | TGGCCTGCTTGCTGTTCTTACAGGGA**[A/G]**GGAGGCAATGGCGGCCAGCACTTCC |
|  |  |  | rs4334089 | intron 2 |  | G | A | TCTCCACCAGGCAGCTCCGGTCCCAT**[A/G]**CACGGCTGATTTGCCCAAACGTACC |
|  |  |  | rs4237855 | intron 2 |  | A | G | AGGACCATCAGTAGTCGAGCTAGAAG**[A/G]**GAGAGGTAAAGGAGAGACCTGAAAA |
|  |  |  | rs7299460 | intron 1 |  | C | T | TCTGGTGGGTTCTGCTTCCTGCTATC**[C/T]**CTCAGAGGGCCTGGAGTCATGCTGG |
|  |  |  | rs4760658 | intron 1 |  | A | G | CCCTAGGACCTGACTGCTGCACAGGC**[A/G]**ACAGCGAGTTTCTAGCAGAGGTTCC |
|  |  |  | rs4516035 | 5' UPST |  | T | C | CGATGACCTCCTTTAGCCAGGGAAGA**[C/T]**ATTGCTATTCGCCTCTTACAGAGGA |
| *GC* refers to Vitamin D binding protein gene; *VDR* refers to Vitamin D receptor gene.  Bold in sequence context denotes nucleotide change.  Location is described considering as the first Exon 1 of consensus sequence. DWST means downstream, UTR means untranscribed region and UPST means upstream. | | | | | | | | |
