# Supplementary material for: rs12512631 on the Group Specific Complement (Vitamin D-Binding Protein GC) Implicated in Melanoma Susceptibility
Source: PLoS One. 2013 Mar 27;8(3):e59607. doi: 10.1371/journal.pone.0059607 (PMC3609832; doi:10.1371/journal.pone.0059607)
Supplement: Table S2 — Case-control study based on haplotypes in the GC gene conducted in Spanish population. Bold denotes statistically significant results. Italic Haplotypes on each block mark the risk haplotype. The Marker number indicates the order of the tag-SNP on the gene. LD Block means linkage disequilibrium block. Frequencies are calculated for the association alleles of each haplotype among 530 cases and 314 controls. (DOCX) [file pone.0059607.s002.docx]

| **Table S2.** Case-control study based on haplotypes in the *GC* gene conducted in Spanish population. | | | | | | | |
| --- | --- | --- | --- | --- | --- | --- | --- |
| **SNP** | **Marker** | **LD Block** | **Haplotypes** | **Frequencies** | | **Chi square** | **p-value** |
|  |  |  |  | **Cases** | **Controls** |  |  |
| rs12512631 | 1 | Block 1 | T | 0.628 | 0.691 | 6.819 | **9x10^-3^** |
|  |  |  | *C* | 0.372 | 0.309 | 6.819 | **9x10^-3^** |
| rs222049 | 2 | Block 2 | *CCGG* | 0.528 | 0.535 | 0.073 | 0.788 |
| rs705119 | 3 |  | CAAT | 0.285 | 0.295 | 0.161 | 0.688 |
| rs4588 | 4 |  | *CAGT* | 0.105 | 0.068 | 6.473 | **0.011** |
| rs7041 | 5 |  | GAGT | 0.033 | 0.056 | 5.172 | **0.023** |
|  |  |  | GCGG | 0.030 | 0.027 | 0.135 | 0.713 |
| rs188812 | 6 | Block 3 | AAT | 0.552 | 0.581 | 1.296 | 0.255 |
| rs222016 | 7 |  | AAC | 0.276 | 0.306 | 1.685 | 0.194 |
| rs1155563 | 8 |  | *TGT* | 0.069 | 0.071 | 0.019 | 0.890 |
|  |  |  | *AGT* | 0.059 | 0.035 | 4.663 | **0.031** |
| rs1352844 | 9 | Block 4 | *C* | 0.880 | 0.896 | 0.945 | 0.331 |
|  |  |  | T | 0.120 | 0.104 | 0.945 | 0.331 |
| rs1352845 | 10 | Block5 | *AG* | 0.807 | 0.842 | 3.117 | 0.078 |
| rs3733359 | 11 |  | GG | 0.156 | 0.126 | 2.769 | 0.096 |
|  |  |  | AA | 0.030 | 0.017 | 2.841 | 0.092 |
| Bold denotes statistically significant results. Italic Haplotypes on each block mark the risk haplotype.  The Marker number indicates the order of the tag-SNP on the gene.  LD Block means linkage disequilibrium block.  Frequencies are calculated for the association alleles of each haplotype among 530 cases and 314 controls. | | | | | | | |
